# Transcriptome analyses to investigate symbiotic relationships between marine protists

**DOI:** 10.3389/fmicb.2015.00098

**Published:** 2015-03-17

**Authors:** Sergio Balzano, Erwan Corre, Johan Decelle, Roberto Sierra, Patrick Wincker, Corinne Da Silva, Julie Poulain, Jan Pawlowski, Fabrice Not

**Affiliations:** ^1^UMR 7144, Université Pierre et Marie Curie Université Paris 06, Sorbonne Universités, Station Biologique de RoscoffRoscoff, France; ^2^Centre National de la Recherche Scientifique, UMR 7144, Station Biologique de RoscoffRoscoff, France; ^3^Centre National de la Recherche Scientifique and Université Pierre et Marie Curie, FR2424, ABiMS, Station BiologiqueRoscoff, France; ^4^Department of Genetics and Evolution, University of GenevaGeneva, Switzerland; ^5^Commissariat à l'Energie Atomique et aux Energies AlternativesGenoscope, France

**Keywords:** radiolarian, ESTs, c-type lectins, plankton, photosymbiosis, Rhizaria

## Abstract

Rhizaria are an important component of oceanic plankton communities worldwide. A number of species harbor eukaryotic microalgal symbionts, which are horizontally acquired in the environment at each generation. Although these photosymbioses are determinant for Rhizaria ability to thrive in oceanic ecosystems, the mechanisms for symbiotic interactions are unclear. Using high-throughput sequencing technology (i.e., 454), we generated large Expressed Sequence Tag (EST) datasets from four uncultured Rhizaria, an acantharian (*Amphilonche elongata*), two polycystines (*Collozoum* sp. and *Spongosphaera streptacantha*), and one phaeodarian (*Aulacantha scolymantha*). We assessed the main genetic features of the host/symbionts consortium (i.e., the holobiont) transcriptomes and found rRNA sequences affiliated to a wide range of bacteria and protists in all samples, suggesting that diverse microbial communities are associated with the holobionts. A particular focus was then carried out to search for genes potentially involved in symbiotic processes such as the presence of c-type lectins-coding genes, which are proteins that play a role in cell recognition among eukaryotes. Unigenes coding putative c-type lectin domains (CTLD) were found in the species bearing photosynthetic symbionts (*A. elongata, Collozoum* sp., and *S. streptacantha*) but not in the non-symbiotic one (*A. scolymantha*). More particularly, phylogenetic analyses group CTLDs from *A. elongata* and *Collozoum* sp. on a distinct branch from *S. streptacantha* CTLDs, which contained carbohydrate-binding motifs typically observed in other marine photosymbiosis. Our data suggest that similarly to other well-known marine photosymbiosis involving metazoans, the interactions of glycans with c-type lectins is likely involved in modulation of the host/symbiont specific recognition in Radiolaria.

## Introduction

Marine planktonic microorganisms are major constituents of oceanic ecosystems, being responsible for half of global primary production and participating massively to carbon sequestration to the deep ocean (Falkowski and Raven, 2013). Despite its recognized global impact, plankton represents one of the least explored compartments of the biosphere, particularly with regards to biotic interaction such as symbiosis. Radiolaria are abundant and widespread unicellular plankton, their large cells, typically ranging from 0.1 to 1 mm in size, exhibit mineral skeletons made up either of silica for Polycystinea or strontium sulfate for Acantharia. Radiolaria can account for a significant proportion of plankton communities in the water column (Dennett et al., 2002; Nimmergut and Abelmann, 2002) and are considered active predators (Swanberg and Caron, 1991). A number of radiolarian species exhibit also a mixotrophic metabolism, hosting eukaryotic photosymbiotic microalgae such as dinoflagellates (Gast and Caron, 1996), chlorophytes (Gast et al., 2000), and haptophytes (Decelle et al., 2012a). The intracellular algae provide photosynthetic products to the host which in turn creates a nutrient-rich micro-environment facilitating algal growth, the symbiotic interactions turning out to be critical for marine food webs and biogeochemical processes (Stoecker et al., 2009). Protists without a clear role as photosymbionts may also be present within radiolarian cells, as found for the acantharian *Acanthochiasma* sp. (Decelle et al., 2012b). Similarly marine invertebrates such as corals, anemones and sponges are known to harbor diverse microbial communities along with their photosymbionts (Radax et al., 2012; Sun et al., 2014).

Well-known photosymbiotic interactions in the marine environment include those occurring between Cnidaria and dinoflagellate from the genus *Symbiodinium* (La Jeunesse, 2001; Schwarz et al., 2008; Weis, 2008), and to a lesser extent those involving sponges (La Jeunesse, 2001) and benthic Foraminifera (Lee, 2006). Little is known about the interaction mechanisms between the host and the symbionts: lectin/glycan interactions were found to be involved in host/symbiont recognitions for different cnidarian taxa (Wood-Charlson et al., 2006; Vidal-Dupiol et al., 2009; Wood-Charlson and Weis, 2009). c-Type lectins are carbohydrate binding proteins involved in a range of different functions including cell adhesion, pathogen recognition and phagocytosis (Zelensky and Gready, 2005). Although a role of c-type lectins in symbiosis has been demonstrated for metazoa (Meyer and Weis, 2012) and to a lesser extent fungi (Singh and Walia, 2014), these proteins are present in a range of organisms and genome comparison based on hidden-Markov models suggest that putative c-type lectins are also present in several protists (http://supfam.org/SUPERFAMILY/cgi-bin/taxviz.cgi?sf=56436). Planktonic Rhizaria are essentially uncultured and tedious to collect, making investigations of symbiotic interactions difficult. Consequently, the mechanisms for host/symbiont recognition in Rhizaria are essentially unknown. In this context, our primary objective was to provide a general overview of the genes occurring in these species for which genomic data are not available and to identify genes potentially involved in symbiotic processes. To achieve our goal we produced and compared large Expressed Sequence Tag (EST) datasets from three photosymbiotic Radiolaria, an acantharian (*Amphilonche elongata*) and two polycystines (*Collozoum* sp. and *Spongosphaera streptacantha*), and one heterotrophic Rhizaria, the phaeodarian *Aulacantha scolymantha* (Cercozoa).

## Materials and methods

### Sample collection

The Phaeodaria (Cercozoa) *A. scolymantha*, and the Polycystinea (Radiolaria) *Collozoum* sp. and *S. streptacantha* were collected with a plankton net in the bay of Villefranche-sur-mer (43°41 N, 7°18 E, Mediterranean Sea) whereas the Acantharia *A. elongata* was collected in the Gulf of Eilat (29°32 N, 34°57 E, Red Sea).

The specimens were sorted manually from the plankton mix using a stereomicroscope by pipetting out of the samples, one by one, the cells of interest. The taxonomically homogeneous pool of sorted cells (one colony was isolated for *Collozoum* sp., about 150 cells for *A. elongata*, and 50 cells for both *A. scolymanta* and *S. streptacantha*) were maintained at least 2 h (up to 8 h) in a petri dish filled with 0.22 μm-filtered seawater. Since digestion of preys usually occurs within the time frame of minutes (Not personal observation) this procedure allowed minimizing sample contamination by marine microorganisms other than those closely associated with the sorted organisms. Cells were then collected individually from the petri dish and rapidly transferred (ca. 1 min) through two successive rinsing steps in clean, 0.22 μm-filtered seawater, to a sterile 1 mL tube. RNA*later* was added to the tube which was stored overnight at 4°C and then transferred to −80°C until further analysis.

### RNA isolation, cDNA library preparation and sequencing

Total RNA was isolated from the samples using the NucleoSpin XS (Macherey-Nagel, Germany) following the manufacturer instructions. Immediately after RNA isolation, cDNA synthesis and amplification was performed using the SMARTer RACE cDNA amplification kit (TaKaRa BIO/Clontech, Mountain View, CA) followed by the library preparation protocol available on Matzlab website (http://www.bio.utexas.edu/research/matz_lab/). Approximately 2.5 μg of the cDNA pool from prepared libraries were used for a titration run using one-quarter of a plate for each sample on the Roche 454 Genome Sequencer FLX using GS-FLX Titanium series reagents. Sequencing was carried out at Genoscope Institute (Evry, France). Data have been submitted to the GenBank under the Sequence Read Archive (SRA) numbers SRR1734678 (*A. elongata*), SRR1744093 (*Collozoum* sp.), SRR1734679 (*S. streptacantha*), and SRR1734688 (*A. scolymantha*).

### Assembly, data cleaning, and annotation

Raw 454 reads were cleaned and assembled using Newbler rd454_mapasm_08172010 (454.com/products/analysis-software). Sequencing adaptors were trimmed from the dataset using the –vt option and reads were finally assembled in contigs using the Newbler option -cDNA, which specifies a cDNA based assembly project. All sequences shorter than 200 bp were removed from the dataset. Seqclean (https://sourceforge.net/projects/seqclean) was then used to remove sequences of low quality. Overall the number of contigs recovered was 3252 for *A. elongata*, 4888 for *Collozoum* sp., 3229 for *S. streptacantha*, and 3243 for *A. scolymantha* (Table 1).

**Table 1:** **Statistics of the EST raw reads and assembly**.

		***A. elongata***	***Collozoum* sp**.	***S. streptacantha***	***A. scolymantha***	**Total**
Total sequenced	Raw reads	195,890	214,475	220,239	195,070	825,674
	Cleaned reads	138,867	121,079	120,924	126,494	507,364
	BP	5.0 × 10^7^	4.2 × 10^7^	4.2 × 10^7^	4.1 × 10^7^	1.7 × 10^8^
	Average length	362	337	348	369	
	Median length	365	324	338	378	
After assembly	Unigenes	14,210	42,784	25,590	42,251	124,835
	BP	6.5 × 10^6^	1.6 × 10^7^	1.0 × 10^7^	1.7 × 10^7^	4.9 × 10^7^
	GC %	46.3	44.0	50.9	40.6	
	Coverage	8.6	3.3	5.2	3.2	
Contigs	No	3252	4888	3229	3243	14,612
	Assembly size (BP)	2.8 × 10^6^	3.4 × 10^6^	2.8 × 10^6^	2.9 × 10^6^	1.2 × 10^7^
	%BP assembled	42.4	21.6	26.9	16.9	
	Average length	858	699	883	893	
	Median length	663	544	606	637	
Annotations	Blastn	410	153	1486	409	2458
	Blastx	13,800	42,631	24,104	41,842	122,377
	Unigenes annotated to GO	2798	4839	4662	6938	19,237
	GO terms	2002	3027	3239	4318	6077
	Unigenes annotated to KEGG	1303	1665	2264	2047	7279
	KEGG pathways	98	101	105	111	123

Contigs and singletons associated to rRNA were identified and extracted from the dataset by a blastn similarity search (Altschul et al., 1997) against the NCBI-nt database and the following keywords were searched within the description line of the blast results: 5.8S, 16S, 18S, 23S, 26S, 28S, ITS, rRNA, rDNA, ribosomal DNA, ribosomal RNA, LSU, and SSU. Reads matching with reference sequences containing one of those keywords, sharing >80% identity with them, and an alignment length >100 bp were considered as ribosomal sequences. The remaining sequences were further screened for the presence of rRNA reads as described in Radax et al. (2012). Unigenes were blasted against both LSU and SSU databases (releases 115, http://www.arb-silva.de/download/archive) (Quast et al., 2013) using the SILVA NGS platform (https://www.arb-silva.de/ngs/) and no additional sequences related to rRNA were found in the in the dataset. The remaining sequences were then considered as mRNA and annotated by homology using blastx similarity search against the NCBI –nr database (version 04/2012).

The taxonomic composition of both ribosomal and non-ribosomal sequences was inferred from the blast results using Megan (Huson and Mitra, 2012). The GC ratio distribution from the different sequences of our putative mRNA dataset was inferred using a homemade python script (github.com/frederic-mahe/bioinformatics-scripts/blob/master/GC_content.py).

### Gene ontology (GO) term distribution and metabolic pathways

Gene ontology terms were assigned to unigenes against the non-redundant protein database NR (April 2012) using the Blast2GO tool (Conesa et al., 2005). Overall our EST were assigned to 6264 different GO terms (Supplementary Table S1) which were then grouped into 117 categories (Supplementary Table S2) using the web-based tool CateGOrizer (Zhi-Liang et al., 2008) with the GO_slim classification method (http://geneontology.org/page/go-slim-and-subset-guide). To reconstruct the known metabolic pathways occurring in our samples, the assembled unigenes were annotated with corresponding enzyme commission (EC) numbers against the Kyoto Encyclopedia of Genes and Genomes (KEGG) database (Kanehisa and Goto, 2000) using the Blast2Go program (http://www.blast2go.com/b2ghome).

### Clustering

Similarity between our different samples was evaluated by clustering the four mRNA libraries. Sequences were translated into the six possible reading frames and clustered using Cd-hit (Li and Godzik, 2006) with 50% sequence identity as described in Toseland et al. (2013). Clusters were examined and sorted using a Ruby script (https://github.com/georgeG/bioruby-cd-hit-report). A Venn diagram was constructed to compare the number of clusters shared by each specimen, using the VennDiagram package with the R version 2.15.0 (http://cran.r-project.org).

### Symbiosis-related genes search

To evaluate the occurrence of ESTs likely coding for symbiosis-related proteins, keyword searches were carried out on the description lines of both blastx and GO results. Enzymes and receptors involved in different functions to the interactions between cnidaria and dinoflagellates were searched. Specifically we searched for genes overexpressed in the coral *Acropora palmata* while in symbiosis with *Symbiodinium* spp., compared to non-symbiotic stages of the same species (Iversen and Seuthe, 2011). Similarly we analyzed the occurrence of candidate symbiosis genes which were previously found in different anemone and coral species (Meyer and Weis, 2012). In coral/dinoflagellate symbiosis these genes are involved in several functions such as pattern recognition, cell adhesion, vesicular trafficking, regulation of incoming light, apoptosis, nutrient and metabolite transport, lipid storage and transport, and response to oxygen reactive species (Meyer and Weis, 2012).

### c-type lectin extraction and phylogenetic analyses

The presence of genes putatively expressing c-type lectins was evaluated by a tblastn similarity search of all the six possible reading frames of the four samples mRNA against a selection of c-type lectins domains downloaded from Genbank using Geneious software (Drummond et al., 2011). The lectins used in this search were derived from the scleractinian corals *Pocillopora damicornis* (Vidal-Dupiol et al., 2009) and *Acropora millepora* (Kvennefors et al., 2008), the sea anemone *Nematostella vectensis* (Wood-Charlson and Weis, 2009), and the nematodes *Laxus oneistus* and *Stilbonema magnum* (Bulgheresi et al., 2011). Several unigenes from *S. streptacantha*, and two unigenes from both *A. elongata* and *Collozoum* sp. showed similarities with reference c-type lectin domains. All but one unigene extracted from our *S. streptacantha* library contained several different putative c-type lectin domains. According to previous studies (Zelensky and Gready, 2005) we define a c-type lectin domain (CTLD) as a sequence containing four conserved cysteine motifs and WIGL-like regions. Unigenes from *S. streptacantha* containing multiple CTLD were then split in the different domains and all the 28 domains obtained from *S. streptacantha* were then aligned to check similarity and remove redundant sequences. We finally obtained eight unique c-type lectin domains from *S. streptacantha*, which were aligned with putative c-type lectin domains from *A. elongata, Collozoum* sp. as well as reference c-type lectins from marine symbiosis described above and seven putative c-type lectins from other protists such as Apicomplexa (*Cryptosporidium parvum, Cryptosporidium muris, Gregarina niphandrodes, Neospora canicum*), Pelagophyceae (*Aureococcus anophagefferens*), Cryptophyta (*Guillardia theta*), and Foraminifera (*Reticulomyxa filosa*). Sequences were aligned using clustal omega (Sievers et al., 2011) and the alignment was cured using gblocks (Castresana, 2000). The final alignment contained 43 sequences with 246 unambiguously aligned positions, phylogenetic inferences were conducted using phyml (Guindon et al., 2010). The maximum likelihood results are shown as an unrooted tree.

## Results

### Assemblage quality

**Figure 1:**
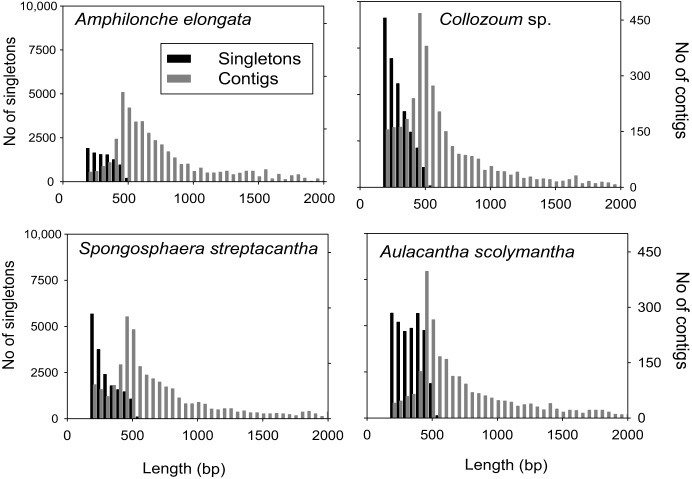
**Distribution of contig and singleton lengths for *A. elongata, Collozoum* sp., *S. streptacantha*, and *A. scolymantha***. Contigs longer than 2000 bp (*n* = 139 for *A. elongata*, max length 2400 bp, *n* = 220 for *A. scolymantha*, max length 11,307, *n* = 110 for *Collozoum* sp., max length 4000, *n* = 75 for *S. streptacantha*, max length 30,120) are not shown.

A total of 825,674 raw reads were generated, and after removal of bad quality and short (<200 bp) sequences we obtained 507,364 reads for a sequencing size of 1.7 × 10^8^ bp (Table 1). Both the number of reads and the sequencing size did not differ significantly (<20%) between the different holobionts. Reads assembled using Newbler led to a number of unigenes (contigs + singletons) from 14,210 (*A. elongata*) to 42,784 (*Collozoum* sp.). *Collozoum* sp. yielded the highest number of contigs (4888) and largest assembly size (3.4 × 10^6^ bp). *A. elongata* ESTs were the best assembled in terms of coverage (8.1X) and proportion of bp assembled (42.4%, Table 1), whereas *A. scolymantha* and *Collozoum* sp. holobionts were the most poorly assembled with a coverage of 3.2X and 3.3X and 21.9 and 26.6% of bp assembled, respectively (Table 1). Most (93–97%) of our contigs were ≤2000 bp, and the median contig length ranged from 544 (*Collozoum* sp.) to 663 (*A. elongata*, Table 1) bp and the contig length peaked at 500 bp for all the samples (Figure 1).

### Ribosomal rRNA

All the unigenes obtained were blasted against the GenBank nucleotide database (blastn) to discriminate between rRNA and putative mRNA sequences. Blastn hits from rRNA reads included both eukaryotic (18S, ITS, and 28S rRNA) and as prokaryotic (16S, ITS and 23S rRNA) genes (Table 2). The number of rRNA unigenes ranged from 153 (*Collozoum* sp.) to 1406 (*S. streptacantha*). Among rRNA unigenes 10% (*S. streptacantha)* to 33% (*A. elongata*) were affiliated to prokaryotes. Prokaryotic unigenes were dominated by γ-Proteobacteria and Bacteroidetes, with α-Proteobacteria and Planctomycetes also frequently occurring throughout our samples (Table 2).

**Table 2:** **Taxonomic distribution of ribosomal (rRNA) and messenger RNA (mRNA) reads^a^**.

	***A. elongata***		***Collozoum* sp**.		***S. streptacantha***		***A. scolymantha***	
	**rRNA**	**mRNA**	**rRNA**	**mRNA**	**rRNA**	**mRNA**	**rRNA**	**mRNA**
α-Proteobacteria	2	7	1	47	5	30	8	19
β-Proteobacteria	0	11	3	15	0	6	2	5
γ-Proteobacteria	135	1645	16	242	53	1315	44	255
δ/ε-Proteobacteria	0	12	8	220	2	24	1	39
Unidentified	0	45	0	139	0	121	0	130
Total Proteobacteria	137	1720	28	663	60	1496	55	448
Bacteroidetes	0	22	5	167	2	51	9	304
Planctomycetes	0	0	2	5	1	0	7	11
Cyanobacteria	1	0	0	32	1	20	1	21
Firmicutes	0	32	2	169	0	38	2	178
Actinobacteria	0	26	3	39	0	53	2	52
Chlamidiae	0	0	0	24	0	0	3	23
Unidentified bacteria	0	277	0	824	0	575	1	999
Other	0	0	2	20	1	21	5	6
**Total bacteria**	**138**	**2077**	**42**	**1943**	**65**	**2254**	**85**	**2042**
Acantharea	101	0	9	0	49	66	16	0
Cercozoa	32	0	2	0	119	0	28	7
Foraminifera	13	47	1	0	4	0	0	0
Polycystinea	12	0	10	0	521	0	2	0
Sticholonchida	10	0	1	0	7	0	0	0
Unclassified Rhizaria	3	7	2	100	93	66	1	103
Gromiidae	0	0	0	0	0	0	4	0
**Total Rhizaria**	**171**	**54**	**25**	**100**	**793**	**132**	**51**	**110**
Apicomplexa	12	37	2	231	3	48	3	289
Dinophyceae	3	35	45	43	3	0	20	15
Perkinsea	0	6	0	40	0	42	0	49
Ciliophora	7	82	1	278	9	108	0	305
Unidentified	0	14	0	40	0	24	0	31
**Total Alveolata**	**22**	**174**	**48**	**632**	**15**	**222**	**23**	**689**
Blastocystae	0	10	0	13	0	15	0	25
Diatoms	7	66	4	100	4	66	3	103
Developayella	0	0	0	0	2	0	1	0
Dictyochophyceae	0	0	0	0	2	0	0	0
Labyrinthulida	0	0	0	0	0	0	4	20
Oomycetes	0	34	0	171	1	78	15	225
Pelagophyceae	0	77	0	122	0	137	0	217
PX clade	0	22	0	81	3	44	2	107
Synurophyceae	0	0	1	0	0	0	0	0
Unidentified Heterokonts	0	38	0	127	0	85	0	155
**Total Heterokonts**	**7**	**247**	**5**	**614**	**12**	**425**	**25**	**852**
Haptophyta	9	28	0	0	0	0	2	6
Cryptophyta	4	0	0	0	1	0	0	6
Excavata	0	65	17	261	1	78	0	269
Fungi	7	187	2	947	82	424	31	777
Hyphochytriomycetes	0	0	0	0	3	0	1	0
Metazoa	19	756	1	3101	42	2457	162	5730
Choanoflagellates	0	46	0	130	0	96	0	534
Unidentified Opisthokonta	0	346	0	1077	0	769	0	1675
Amoebozoa	2	64	0	209	0	84	2	578
Rhodophyta	2	0	0	0	0	0	0	0
Chlorophyta	2	91	2	0	31	137	1	2
Streptophyta	6	209	0	0	6	421	0	2
Unidentified Archaeoplastida	0	36	0	996	0	93	0	971
**Total Archaeoplastida**	**10**	**336**	**2**	**996**	**37**	**651**	**1**	**975**
Unidentified eukaryotes	19	2285	3	7040	134	4349	20	6523
**Total eukaryotes**	**270**	**4588**	**103**	**15,107**	**1120**	**9687**	**318**	**18,724**
**Archea**	**0**	**0**	**0**	**0**	**0**	**0**	**0**	**11**
**Virus**	**0**	**0**	**0**	**10**	**0**	**29**	**0**	**12**
**No hits**	**0**	**5782**	**0**	**22,583**	**0**	**10,238**	**0**	**18,303**
**Not assigned**	**0**	**1353**	**0**	**2988**	**0**	**1962**	**0**	**2754**
**Total**	**408**	**13,800**	**145**	**42,631**	**1185**	**24,170**	**403**	**41,846**
**% Rhizaria sequences**	**42**	**0.4**	**17**	**0.2**	**67**	**0.5**	**13**	**0.2**

a *Results were obtained based on blastn and blastx search, respectively*.

Eukaryotic rRNA unigenes included from 13% (*A. scolymantha*) to 71% (*S. streptacantha*) of rhizarian sequences. Hundred and one rRNA unigenes from *A. elongata* were affiliated to several Acantharia genera, with only five unigenes associated with the genus *Amphilonche* (Supplementary Table S3). Polycystinea were poorly represented in the rRNA library of *Collozoum* sp. since only 10 out of 103 eukaryotic rRNA reads shared high similarities with Polycystinea. In contrast, about 25% of rRNA reads from *S. streptacantha* were affiliated to Polycystinea (Table 2), but none of them could be affiliated to the genus *Spongosphaera*, very likely because reference sequences from this genus are missing in GenBank. Rhizarian rRNA unigenes from *A. scolymantha* were mostly affiliated with Cercozoa (Table 2), with only one out of 28 cercozoan unigenes assigned to *A. scolymantha* (Supplementary Table S3).

Other eukaryotic sequences recovered within the rRNA from our holobionts were mainly affiliated to Alveolata (Table 2), specifically Apicomplexa for *A. elongata*, ciliates for *S. streptacantha* and dinoflagellates for *Collozoum* sp. and *A. scolymantha*. rRNA reads from known acantharian symbionts such as *Phaeocystis* sp. (Decelle et al., 2012a) were found for *A. elongata* holobionts along with reads from other photosynthetic protists such as Bacillariophyceae, Prasinophyceae, and Cryptophyceae (Supplementary Table S3). *S. streptacantha* holobiont also contained >100 microalgal rRNA reads associated with the *Lotharella* sp. (Chlorarachniophyceae) and some reads from Bacillariophyceae and Cryptophyceae.

### Putative mRNA: taxonomic composition

Putative mRNA represented the majority of our dataset, corresponding to 97% of the total number of unigenes for *A. elongata*, 99.7% for *Collozoum* sp., 88% for *S. streptacantha*, and 98.3% for *A. scolymantha*. The putative mRNA unigenes correspond to transcripts occurring in our holobionts at the time of sampling and likely derive from the hosts, symbionts and/or other microorganisms present within and around the rhizarian cells. Overall we blasted 122,377 putative mRNA unigenes against the GenBank protein database (blastx) to obtain a general annotation of the expressed genes and to assign a putative metabolic function based on similarity. Most of the mRNA unigenes matched eukaryotic sequences or did not have significant hits with known reference sequences (no hits). The most represented matches were associated with land plants (Streptophyta), Metazoa and Fungi (Table 2), very likely reflecting the most important occurrence of genomes from these groups in current databases. The scarcity of genomic sequences from rhizarian groups other than Foraminifera and Cercozoa (i.e., no genomes of Polycystinea) implied that *A. elongata, Collozoum* sp. and *S. streptacantha* libraries included a number of transcripts blasting with Foraminifera. A few *A. scolymantha* unigenes were related to mRNA sequences from the cercozoan *Bigelowiella* sp. A minor proportion of the unigenes were assigned to Bacteria (Table 2).

### GC content

We analyzed the GC content of our samples to assess whether our holobionts showed a plurimodal distribution reflecting the occurrence of different microorganisms. The overall GC content of our samples ranged from 40.6% for *A. scolymantha* to 50.9% for *S. streptacantha* (Table 1). We then compared, within each of our holobionts, the GC-content distribution from the different domain level groups (Bacteria, Eukaryotes, no-hits, and not-assigned) and found that the GC content from our bacterial mRNA unigenes differed from that of the eukaryotic unigenes, especially for *S. streptacantha* (Supplementary Figure S1).

**Figure 2:**
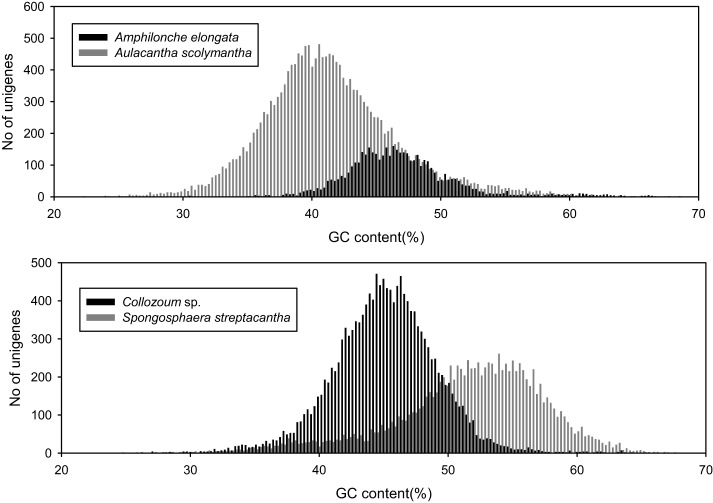
**GC content of putative eukaryotic mRNA unigenes for the four specimens analyzed in the present study**.

However the GC-content of the eukaryotic mRNA from our holobionts showed a unimodal distribution and we could not discriminate the contribution of the different microorganisms within our libraries. Significant differences occurred between the different samples, including the taxonomically closest ones, our two polycystinean species, with most unigenes from *Collozoum* sp. having a lower GC content than the majority of *S. streptacantha* reads (Figure 2).

### Gene ontology and KEGG

**Figure 3:**
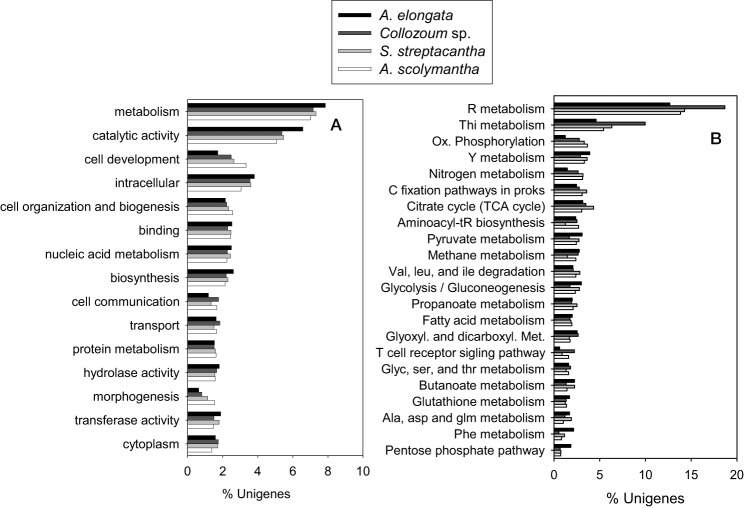
**Comparison of the (A) Gene ontology terms summarized into Goslim categories and (B) KEGG assignments from the transcriptomes of the holobionts analyzed in the present study**.

Overall 19,237 unigenes from our mRNA dataset were annotated to a total of 6264 different GO terms which were then grouped into 117 GOslim categories (Supplementary Tables S1,S2). The most represented categories were related to catalytic activity and cell components (Figure 3). The proportion of unigenes expressed for each category was similar for the different samples, preventing identification of GO categories under-expressed in our non-photosymbiotic sample (*A. scolymantha*) and thus likely related to photosynthetic activities.

Moreover 1303–2264 unigenes from our different holobionts were also annotated to KEGG and a total of 123 KEGG pathways were identified within our dataset (Supplementary Table S4). The most represented pathways were related to nucleotides, thiamine and nitrogen metabolisms as well as citrate cycle and carbon fixation pathways in prokaryotes (Figure 3). A higher proportion of *Collozoum* sp. unigenes were related to purine and thiamine metabolisms compared to the other holobionts, whereas the proportion of ESTs associated with oxidative phosphorylation and nitrogen metabolisms was significantly lower for *A. elongata*. Consistent with GOslim data we could not find any KEGG pathway associated with non-symbiotic *A. scolymantha* over-represented or under-represented with respect to the other holobionts. Therefore, it was not possible to identify genes or pathways likely associated with photosymbiosis.

### Clustering

**Figure 4:**
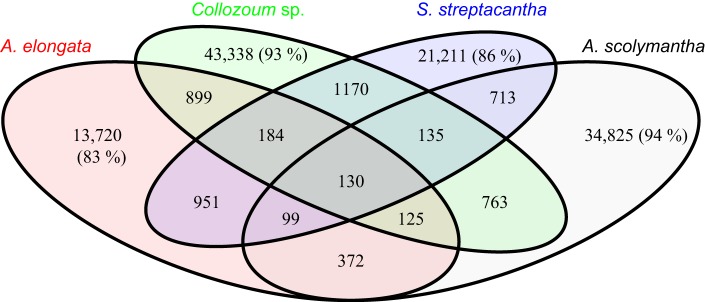
**Venn diagram for pooled sequence clustering based on cd-hit**. A total of 124,979 clusters were obtained using 50% similarity. Values indicate the number of clusters relative to each diagram section. Percentages show portions of clusters which only occur in one specimen.

The majority of the clusters identified here, based on a 50% similarity, only include sequences from one sample (Figure 4). For example 83% of the clusters containing sequences from *A. elongata* do not contain unigenes from other samples (Figure 4), and this percentage rises up to 94% for *A. scolymantha*. Very few clusters thus contain sequences from multiple samples. In spite of the taxonomic relatedness of our specimens only 130 clusters include unigenes from all our samples whereas higher proportions of the clusters are shared between *A. elongata* and *Collozoum* sp. (899), *Collozoum* sp. and *S. streptacantha* (1170), and *A. elongata* and *S. streptacantha* (951) compared to the clusters shared between *A. scolymantha* and any of the three other holobionts (Figure 4). These differences might be related to the lack of photosymbionts within *A. scolymantha*, in contrast with the other samples.

### Symbiosis-related genes and c-type lectin domains

We searched for the presence of genes previously found overexpressed in the symbiotic stages of cnidaria/dinoflagellate holobionts (Kvennefors et al., 2008; Iversen and Seuthe, 2011; Meyer and Weis, 2012; Yuyama et al., 2012). A number of putative mRNA reads from our dataset are related to genes coding glutamate dehydrogenase, glutathione S-transferase, lectins, glutamine synthetase, lipase, phospholipase and ferritin (Supplementary Table S5). Within these genes we examined specifically the occurrence of lectins. Overall 142 unigenes from all our samples shared similarities with lectins (Table 3). Their number ranged from 19 (*A. elongata*) to 57 (*S. streptacantha*). We blasted reference sequences from CTLDs involved in marine symbiosis (Wood-Charlson et al., 2006; Vidal-Dupiol et al., 2009; Bulgheresi et al., 2011) against our dataset of mRNA translated into amino acid sequences in order to identify putative CTLD within our data. Overall eight sequences from the holobionts of *A. elongata* (two), *Collozoum* sp. (two), and *S. streptacantha* (four) showed similarities with CTLDs.

**Table 3:** **Blastx hits for the unigenes related to lectins**.

**Protein type**	***A. elongata***	***Collozoum* sp**.	***S. streptacantha***	***A. scolymantha***
Agglutinin isolectin 1 precursor	0	0	0	1
Cellulose binding elicitor lectin	0	0	2	0
Chitin binding lectin	0	0	1	0
c-type lectin	2	12	6	0
Dextran binding lectin	0	0	0	1
Endoplasmic reticulum lectin	2	1	0	0
Fucolectin	0	0	2	0
Galectin	0	0	1	1
g-type lectin	1	2	1	0
Jacalin related lectins	0	1	2	1
Lactose binding lectin	0	2	0	0
Lectin domain-containing protein	2	0	1	3
Lectin homolog	0	0	0	1
Lectin protein	2	0	0	0
Lectin receptor kinase	0	3	1	0
Lectin type 2	0	0	2	0
Legume lectin β containing protein	0	1	1	3
Mannose binding lectins	4	1	0	5
Rhamnose binding lectin	0	0	0	2
Ricin b lectin	5	1	31	13
Selectin	2	0	0	2
Sialic acid binding lectin	1	1	0	0
Other lectins	0	1	6	5
**Total**	**21**	**25**	**51**	**33**
**% of total blast results**	**0.15**	**0.05**	**0.09**	**0.05**

**Figure 5:**
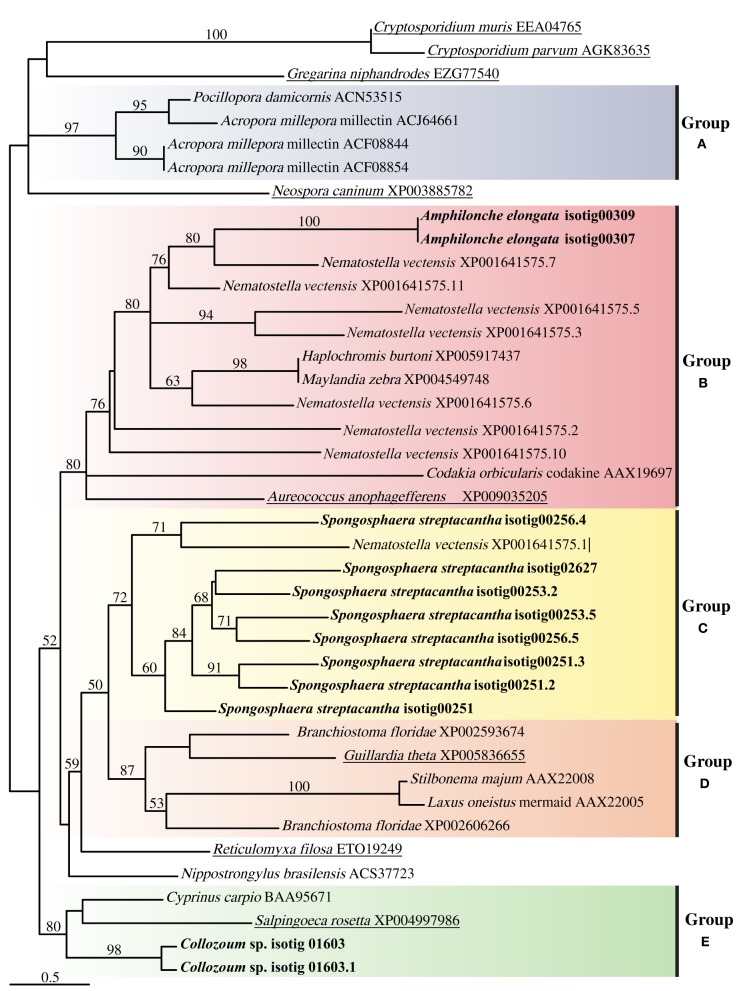
**Unrooted tree describing the relatedness of c-type lectins of radiolarians in the present study, other protists and Metazoa harboring photosynthetic symbionts**. The consensus tree resulting from phyml analyses of 246 unambiguously aligned positions and 43 protein sequences. Topological support of >50% obtained from aLRT approach are shown in each branch label. Sequences corresponding to this study are in bold whereas sequences from other protists are underlined.

The alignment of putative CTLD from our samples with CTLD from other marine holobionts or putative CTLD from other protists consisted of four highly conserved cysteine residues as well as four motifs (Supplementary Figure S3). The WIGL-like motif which is crucial in the identification of CTLD (Zelensky and Gready, 2005) was found to vary for our holobionts, being WIGV for *A. elongata*, WLGF for *Collozoum* sp. and WIGL, WLGL, and WVGL for the different CTLD from *S. streptacantha* (Supplementary Figure S3). The glucose/mannose binding motif EPN (Zelensky and Gready, 2005) was found only in *S. streptacantha* whereas such motif was replaced by DSS in *A. elongata* and by WSD in *Collozoum* sp. Similarly the galactose binding motif LND (Zelensky and Gready, 2005) was only found in *S. streptacantha* CTLD. In *A. elongata* the LND motif was replaced by VND whereas we could not find an equivalent motif in *Collozoum* sp. (Supplementary Figure S3). The resulting phylogenetic tree reconstruction is shown as an unrooted tree (Figure 5), and includes putative CTLD extracted from the holobionts as well as a range of reference sequences from Metazoa and protists. Five well-supported c-lectin groups can be identified from the phylogenetic tree. Group A strictly includes coral CTLDs that could be related to several ungrouped sequences from alveolates. In group B we find the protist CTDLs of *A. elongata* and *A. anophagefferens* more closely related to other metazoans CTDLs than to other acantharians and apicomplexan protists, respectively. Group C is almost exclusively composed by *S. streptacantha* except for a sequence from *N. vectensis*. Group D is an assembly of various metazoan CTDLs with the presence of the protist *G. theta*. Finally, group E contains the CTDLs of *Collozoum* sp. and the lectins of the *Salpingoeca rosetta* and *Cyprinus carpio*.

The phylogeny found here does not reflect the taxonomic position of the species from which the sequences were extracted but depicts the relatedness of the lectin proteins present in the species shown (Figure 5). Several clusters, each including sequences from both protists and Metazoa were indeed found. Putative CTLDs within each holobionts group together forming well-supported clusters (100 support for *A. elongata*, 98 for *Collozoum* sp., and 72 for *S. streptacantha* CTLDs). Contrarily to what would be expected, the putative CTLDs extracted from the radiolarian holobionts showed to be more closely related to other protists or metazoan lectins than to radiolarians themselves (Figure 5).

## Discussion

### Holobiont sequencing

Nowadays, high throughput sequencing approaches are largely applied to investigate the interactions of microbes with multicellular hosts, especially in the marine environment. Such approaches were used to elucidate symbiotic interactions in Cnidaria (Sabourault et al., 2009; Yuyama et al., 2011) and sponges (Radax et al., 2012). In contrast fewer studies investigated interactions when both partners are protists. Recently, EST analyses have been applied to understand the ability of Foraminifera to maintain chloroplasts deriving from previously ingested diatoms (Pillet and Pawlowski, 2013). In our investigation, because of the relatively low sequencing depth, our coverage is not sufficient to infer expression patterns but allows a qualitative assessment of the genes expressed within radiolarian holobionts. Still we observed different coverage between our datasets, with fewest unigenes recovered from the best assembled sample (*A. elongata*), suggesting some variability in transcript diversity between the holobionts analyzed, the lower coverage the higher diversity. Also, the high proportion of unassigned mRNA unigenes (Table 2) might be related to the fact that Rhizaria is the least sequenced eukaryotic super-group to date (Burki and Keeling, 2014; del Campo et al., 2014).

Only, one rhizarian genome, from *Bigelowiella natans* (Chloroarachniophyta), has been fully sequenced to date (Bhattacharya et al., 2013) and other data only include partially sequenced genomes and transcriptomes, mostly from Foraminifera (Burki et al., 2010; Bulman et al., 2011; Habura et al., 2011; Pillet and Pawlowski, 2013; Sierra et al., 2013). In the present study a number of *A. elongata* reads were assigned to Foraminifera likely because of the evolutionary relatedness of Acantharia and Foraminifera (Sierra et al., 2013). These two classes show higher genetic distances from Polycystinea implying that high proportions of reads from *Collozoum* sp. and *S. streptacantha* were not assigned, even at high taxonomic level.

The high proportion of mRNA sequences related to Streptophyta, unidentified Archaeplastida, Fungi, and Metazoa within our eukaryote-assigned transcripts could be explained by some contamination. Yet considering that proportions of rRNA reads affiliated to these taxa does not match with the mRNA assignments, it is also likely that it exists a bias related to the unevenness of taxonomic coverage within the public database (Keeling et al., 2014). As for a number of eukaryotes, a transcriptomic approach provides a valuable alternative to genome sequencing for uncultured protistan taxa. The use of transcriptomics is also particularly suited to study uncultured Radiolaria as their symbionts are mainly dinoflagellates (Gast et al., 2003; Probert et al., 2014), which have an enormous genome size (Lin, 2011) making sequencing very difficult. In the present study, investigations of the holobionts are important because they bring a holistic view of highly complex unicellular organisms living in close contact with diverse microbial communities.

### Microbial communities associated with rhizarian hosts

Although the technique used for the cDNA library preparation targets specifically eukaryotic mRNA because of poly-T primers used which bind to the poly-A region of transcript, eukaryotic and prokaryotic rRNA as well as prokaryotic mRNA genes were recovered in our libraries. Blastn results of our putative ribosomal reads indicate a wide taxonomic diversity within our samples. Ten bacterial genera typically deriving from contamination while extracting nucleic acids (Salter et al., 2014) were present in very low abundances within the rRNA libraries, and mostly for *A. scolymantha*. In particular five to seven rRNA hits from the genera *Mesorhizobium, Rhizobium* and *Flavobacterium* were found in the *A. scolymantha* rRNA library. The impact of potential lab-contaminant to our data was thus minimal, even for *A. scolymantha*, which seems the specimen mostly affected by contamination.

The presence of symbiotic dinoflagellates in several Polycystinea including *Collozoum* (Gast and Caron, 2001; Probert et al., 2014) and that of symbiotic haptophytes in Acantharia (Decelle et al., 2012a,b) is also reflected here by the taxonomic composition of both rRNA and mRNA unigenes, where dinoflagellates and haptophytes accounted for an important portion of the libraries from *Collozoum* sp. and *A. elongata*, respectively (Table 2). In contrast to previous findings for other spumellarian species (Gast and Caron, 1996; Dolven et al., 2007), the *S. streptacantha* isolated here was unlikely to harbor dinoflagellate photobionts, since very few dinoflagellate rRNA sequences were recovered from this specimen. The high proportion of rRNA reads related to the chlorarachniophyte *Lotharella* sp. (Supplementary Table S6) found within our *S. streptacantha* sample suggests the occurrence of chlorarachniophyte photobionts, which have not been reported yet. However, one cannot exclude that *Lotharella* sp. cells might have been occurring in *S. streptacantha* as preys since the latter species has been recorded to predate on ciliates and small flagellates (Matsuoka, 2007). Accordingly a number of rRNA sequences from ciliates were found within our *S. streptacantha* sample (Table 2). Data from our *A. scolymantha* could illustrate an active predatory activity, since the rRNA reads from this specimens are dominated by marine Metazoa but also include reads from other protists (Table 2) which might be associated with preys engulfed by the host before sampling. As the basic ecology of Rhizaria is often elusive, EST analyses can thus be a useful tool to analyze the composition of microbial communities within our samples and also to address food preferences from rhizarian hosts.

EST analyses provided also useful information on the prokaryotic communities associated with our radiolarian hosts although the role of the different taxa found is unknown. The dominant bacterial classes found in the present study, γ-Proteobacteria and Bacteroidetes, are commonly abundant in surface seawater (Biers et al., 2009; Yooseph et al., 2010). Still, both γ-Proteobacteria and Bacteroidetes can dominate the intracellular environment of distinct mixotrophic protists (Martinez-Garcia et al., 2012) as well as particle-attached bacteria (Simon et al., 2002), which would explain their presence in our holobionts. Surprisingly cyanobacteria, mostly affiliated to the genus *Synechococcus* accounted for a very low proportion of our bacterial rRNA, although cyanobacterial endosymbionts were previously reported to occur within the cercozoan genus *Paulinella* (Bodyl et al., 2007; Bhattacharya et al., 2013), and some polycystinean such as *Dictyocoryne* spp. (Foster et al., 2006a,b; Yuasa et al., 2012) and *Spongostaurus* sp. (Foster et al., 2006b).

Overall, the rRNA sequence composition of our dataset suggests the occurrence of a diverse community in close contact or within our holobionts and confirms the photosymbiont identity for both *A. elongata* and *Collozoum* sp. (Decelle et al., 2012a; Probert et al., 2014) whereas the photosymbiotic status of *S. streptacantha* is unclear.

### Specificities of the holobionts analysis

The differences in mRNA composition found here between our different holobionts (Table 2) are corroborated by our clustering data (Figure 4), since most (83–94%) of the clusters obtained from our mRNA unigenes based on 50% similarity include reads from one single species. These differences might be related to both the hosts and the symbionts. On the one hand clustering data suggest a significant genomic diversity between our hosts although they belong to the same phylum. On the other hand the microbial communities associated with our hosts (Table 2) likely expressed different, unrelated genes, contributing to the low similarities in mRNA found here.

The unimodal distribution of the GC content (Figure 2) within our eukaryotic putative mRNA unigenes does not allow discriminating the host-derived ESTs from those derived from other microorganisms. Such discrimination was complicated by the fact that our data likely include transcripts from a range of different eukaryotic microorganisms making the contribution from each taxon to the overall GC distribution complicate to disentangle. In previous studies, where genomic data from both host and symbionts were available, the contributions from each partner to the transcriptome were successfully discriminated based on the GC%. For example the contributions from the distinct partners were successfully discriminated for the symbiotic coral *Acropora tenuis*, because the holobiont transcriptome was compared with the genome of the symbiont-free larva (Kii et al., 2007). In contrast, Radiolaria are uncultivable, which makes more difficult to obtain symbionts-free radiolarian species. In the present study we used *A. scolymantha* as a photosymbiont free species, however the comparison of *A. scolymantha* mRNA with that obtained from the other species did not allow the discrimination between host- and symbiont-derived transcripts for two reasons. First although photobiont-free, *A. scolymantha* is still likely to harbor a diverse microbial assemblage, as suggested by the rRNA composition (Table 2), consisting in a combination of transcripts from the host and multiple symbionts from which the proportion of purely host transcripts is unknown. Second, since *A. scolymantha* is a phaeodarian branching within Cercozoa, it is phylogenetically distant from both Polycystinea and Acantharia (Krabberod et al., 2011; Sierra et al., 2013); therefore host derived transcripts from *A. scolymantha* might have a different GC profile from *A. elongata, Collozoum* sp. and *S. streptacantha*.

### c-type lectins

The alignment of our sequences with CTLDs from marine organisms strongly suggests the occurrence of c-type lectins in our specimens of *A. elongata, Collozoum* sp. and *S. streptacantha* holobionts. c-type lectins have been described controlling the onset of photosymbiosis in several cnidarian (Wood-Charlson et al., 2006; Vidal-Dupiol et al., 2009), nematodes (Bulgheresi et al., 2006), and fungal (Singh and Walia, 2014) species. Although c-type lectins have been mostly described from Metazoa so far (Zelensky and Gready, 2003) we did not find putative CTLD in our *A. scolymantha* holobiont, which contained high proportions of rRNA and mRNA reads affiliated to Metazoa (Table 2). This suggests that the putative c-type lectins found here in *A. elongata, Collozoum* sp. and *S. streptacantha* are unlikely to derive from metazoan preys or debris present within the holobionts but are rather associated with Rhizaria or other microbial eukaryotes present within the *holobionts*.

The presence of unigenes related to c-type lectins among our symbiotic taxa only (i.e., *A. elongata, Collozoum* sp., and *S. streptacantha*) suggests that these proteins could play a role in the symbiotic process. Although a role in functions other than symbiosis cannot be excluded, since c-type lectins were demonstrated to trigger the onset of photosymbiosis in nematodes and corals, they might also play a role in the recognition of the symbionts in some Rhizaria.

### Conflict of interest statement

The authors declare that the research was conducted in the absence of any commercial or financial relationships that could be construed as a potential conflict of interest.
