# Supplementary material for: Transcriptome analyses to investigate symbiotic relationships between marine protists
Source: Front Microbiol. 2015 Mar 17;6:98. doi: 10.3389/fmicb.2015.00098 (PMC4362344; doi:10.3389/fmicb.2015.00098)

Supplementary Figure S3. Sequence alignment for the c-type lectin domains (CTLDs) found in our holobionts as well as other protists and metazoa. Identifiers from sequences retrieved in the present study are in red. The positions conserved in > 50 % of the sequences are highlighted.
Species abbreviations: *A. millepora, Acropora millepora; P. damicornis, Pocillopora damicornis; S. streptacantha, Spongosphaera streptacantha; N. vectensis, Nematostella vectensis; M. zebra, Maylandia zebra; C. carpio, Cyprinus carpio; B. floridae, Branchiostoma floridae; C. orbicularis, Codakia orbicularis; A. anophagefferens, Aureococcus anophagefferens; S. rosetta, Salpingoeca rosetta; N. brasilensis, Nippostrongylus brasilensis; G. theta, Guillardia theta; R. filosa, Reticulomyxa filosa; L. oneistus, Laxus oneistus; S. majum, Stilbonema majum; A. elongata, Amphilonche elongata; N. caninum, Neospora caninum; G. niphandrodes, Gregarina niphandrodes; C. parvum, Cryptosporidium parvum; C. muris, Cryptosporidium muris*

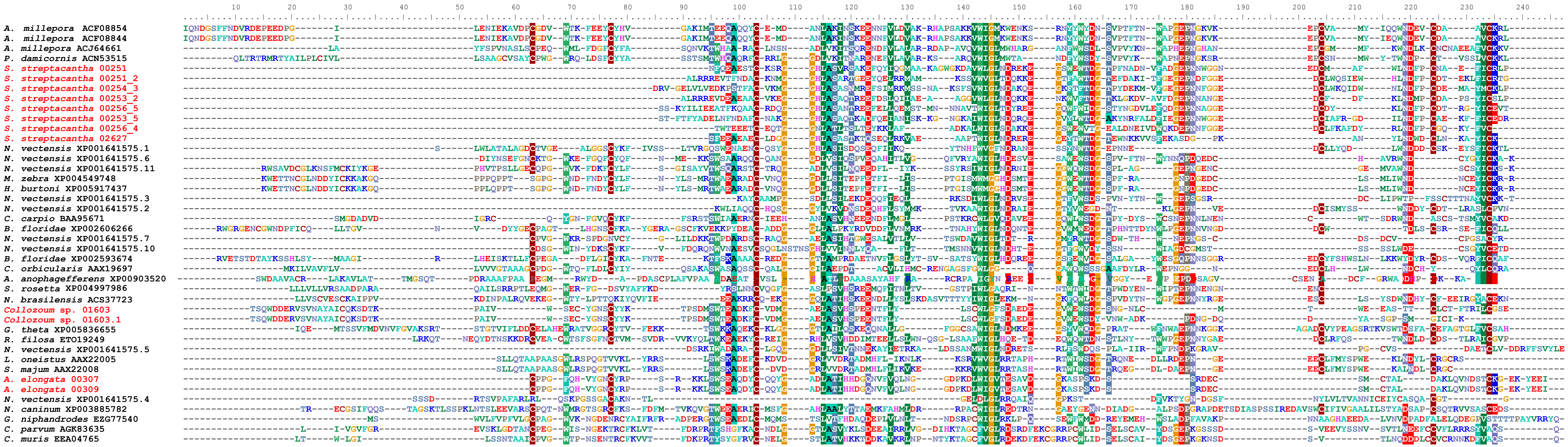

Supplement: Supplementary file 1 [file DataSheet1.ZIP › Supplementary Figure S3.pdf]
