# Supplementary material for: Transcriptome analyses to investigate symbiotic relationships between marine protists
Source: Front Microbiol. 2015 Mar 17;6:98. doi: 10.3389/fmicb.2015.00098 (PMC4362344; doi:10.3389/fmicb.2015.00098)

Supplementary Figure S1. GC content of the different groups of our putative mRNA unigenes detected in the present study. Putative mRNA unigenes were sorted according to the domain (eukaryotic, prokaryotic, no hit and not assigned) based on a blastx search.

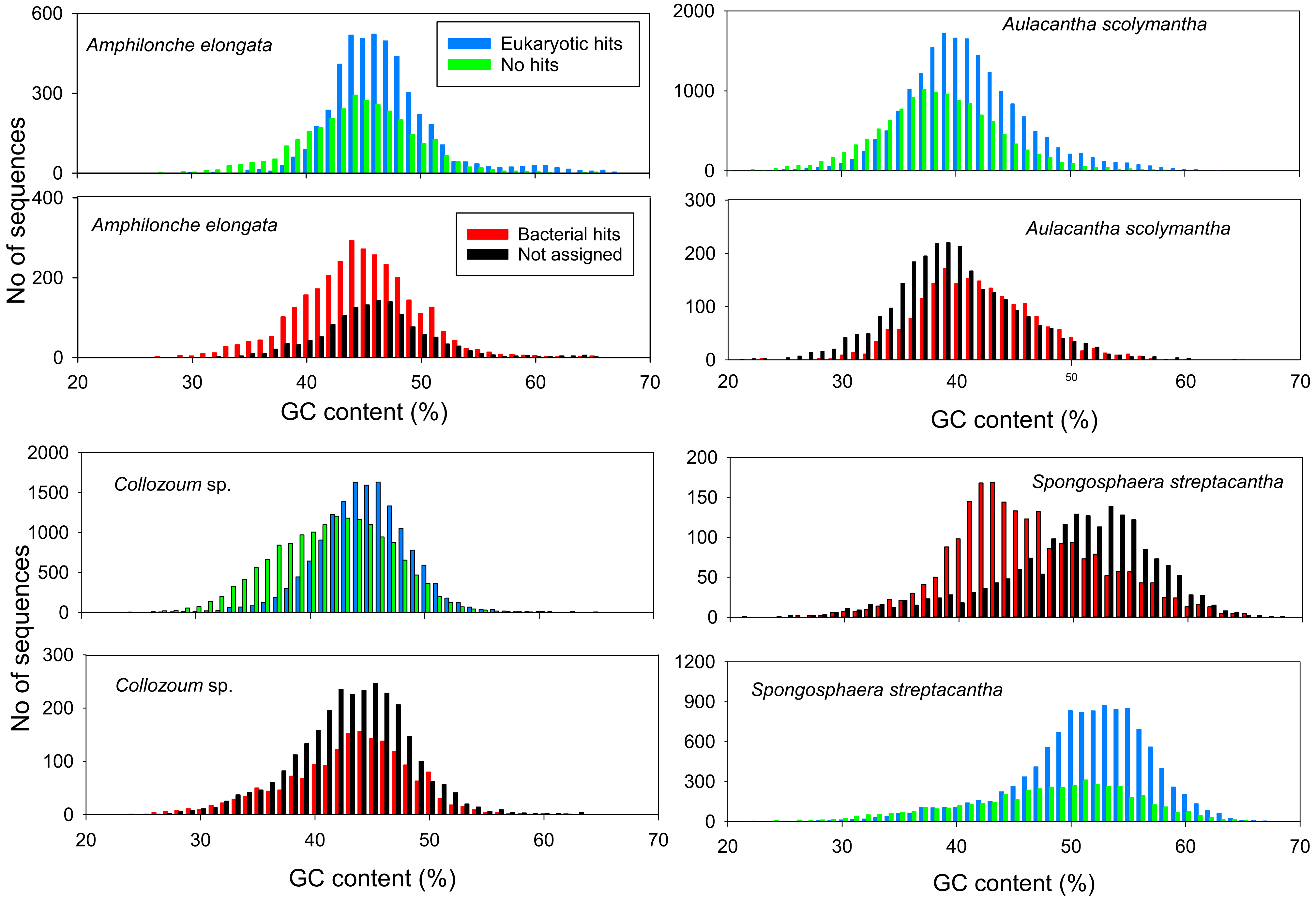

Supplement: Supplementary file 1 [file DataSheet1.ZIP › Supplementary Figure S1.pdf]
