# Supplementary material for: Transcriptome analyses to investigate symbiotic relationships between marine protists
Source: Front Microbiol. 2015 Mar 17;6:98. doi: 10.3389/fmicb.2015.00098 (PMC4362344; doi:10.3389/fmicb.2015.00098)

Supplementary Figure S2. Comparison between assembled and non-assembled ESTs for each different domains. Assemblage quality of non-ribosomal reads after taxonomic (Megan) sorting

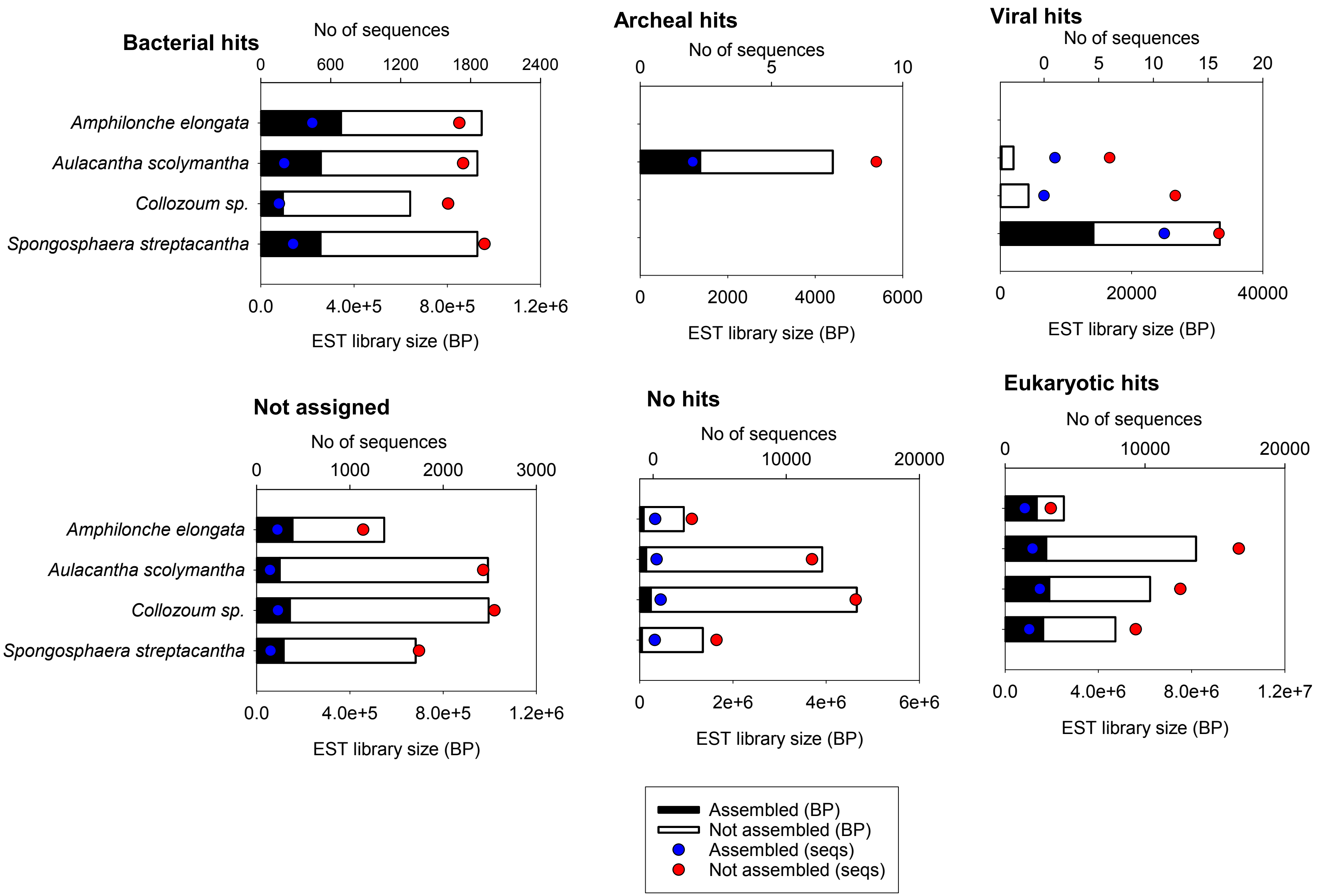

Supplement: Supplementary file 1 [file DataSheet1.ZIP › Supplementary Figure S2.pdf]
